# Integrated analysis of sex differences in human dendritic cell, monocyte, and natural killer cell subsets

**DOI:** 10.3389/fimmu.2026.1750775

**Published:** 2026-03-30

**Authors:** Yanhui Yang, Longxian Chen, Mengwei Shang, Qicong Cao, Mengyang Zhang, Liqin Zhang, Jian Wang, Xin Tong, Xiaoming Sun

**Affiliations:** 1 Zhejiang Key Laboratory of Medical Epigenetics, Department of Immunology and Pathogen Biology, School of Basic Medical Sciences, Hangzhou Normal University, Hangzhou, China; 2 Department of Clinical Laboratory, First People’s Hospital of Linping District, Hangzhou, Zhejiang, China; 3 Ragon Institute of Mass General Brigham, MIT and Harvard, Cambridge, MA, United States; 4 Hangzhou Women’s Hospital, Hangzhou Normal University, Hangzhou, China; 5 State Key Laboratory for Diagnosis and Treatment of Infectious Diseases, The First Affiliated Hospital, College of Medicine, Zhejiang University, Hangzhou, China

**Keywords:** dendritic cells, innate immune responses, monocytes, natural killer cells, sexual dimorphism, subsets

## Abstract

Sex-associated differences in the innate immune system fundamentally influence immune responses, yet subset-specific cellular heterogeneity is often overlooked in population-level studies. To characterize these differences at a granular level, we performed integrated immune profiling—using multiparameter flow cytometry, transcriptomics, and functional assays—on peripheral blood mononuclear cells from 91 healthy mainland Chinese donors. Our analysis revealed significant, conserved sexual dimorphism across major innate immune networks. In dendritic cells (DCs), females possessed a significantly higher frequency of plasmacytoid DCs (pDCs) exhibiting enhanced basal interferon-stimulated gene (ISG) expression and elevated IFN-β production upon stimulation, whereas males showed higher frequencies of Axl^+^ and CD1c^+^ DCs. Furthermore, males exhibited higher overall monocyte frequencies driven by classical monocytes, displaying heightened inflammatory signatures, elevated ICAM-1 protein expression, and the induction of stronger, Th17-skewed CD4^+^ T cell proliferation. Conversely, female monocytes were enriched for antigen presentation pathways, antiviral ISGs, and Th2 T cell polarization. Among natural killer (NK) cells, males demonstrated a higher proportion of mature CD56^dim^ subsets, while females had higher proportions of CD56^bright^ and CD56^neg^ subsets characterized by enhanced signaling, higher expression of activating receptors, and greater secretion of cytotoxic markers. Ultimately, these findings provide a comprehensive, subset-specific map of innate immune sexual dimorphism, demonstrating that female innate immune cells are fundamentally primed for enhanced antiviral interferon responses and robust NK effector functions, whereas male profiles are characterized by heightened inflammatory signatures and cellular maturation.

## Introduction

Biological sex has emerged as a critical variable influencing immunological heterogeneity, with females and males exhibiting distinct differences in immune activation, susceptibility to infections, prevalence of autoimmune diseases, and responses to vaccines (1–4). These differences underscore the importance of dissecting the cellular and molecular drivers of sexual dimorphism to advance research and clinical practice.

This sexual dimorphism is pronounced within the key innate immune cells of peripheral blood—dendritic cells (DCs), monocytes, and natural killer (NK) cells—which collectively influence outcomes in health and disease. Among these, DCs play a pivotal role in bridging innate and adaptive immunity. Females typically exhibit enhanced DC activity, with plasmacytoid DCs (pDCs) producing elevated interferon-alpha via TLR7 signaling, and myeloid DCs (mDCs) generating superior levels of IL-12 and TNF-α. This heightened responsiveness, often fueled by estrogen and X-linked factors like TLR7 and IRF5, strengthens female antiviral defenses against pathogens such as HIV-1, SARS-CoV-2, and West Nile virus, and enhances tumor control in melanoma (5–8). However, this same “ready-to-respond” state amplifies the risk of autoimmune diseases, contributing to the female predominance in diseases like Systemic Lupus Erythematosus (SLE) and asthma (9, 10). In contrast, males often show subdued DC activation and less efficient antigen presentation—potentially due to the suppressive effects of testosterone—which may weaken innate control of pathogens like *Mycobacterium tuberculosis* and influenza (11, 12).

Complementing DCs, monocytes serve as another cornerstone of innate immunity, contributing to phagocytosis and inflammation while exhibiting parallel patterns of sexual dimorphism. Females generally display heightened monocyte activity characterized by increased expression of interferon-related genes and enhanced TLR4 responsiveness (5, 13, 14). While this profile boosts antiviral defense, it further elevates autoimmune risk; for instance, activated monocytes are a hallmark of SLE pathogenesis in women (14, 15). Conversely, males typically exhibit higher circulating monocyte counts and a more robust pro-inflammatory cytokine production (e.g., TNF-α, IL-1B) following bacterial stimulation, a trait modulated by testosterone and larger splenic reserves (16, 17). These patterns manifest clinically in conditions like obesity, Parkinson’s disease, and cardiovascular disorders, where male monocytes drive metabolic inflammation, whereas females favor IFNγ-mediated responses (18, 19).

Similarly, NK cells exemplify profound sexual dimorphism. Females consistently display enhanced functional activity, marked by greater IFNγ production, degranulation (e.g., CD107a), and activation receptor expression (e.g., NKp46, NKG2D), driven by estrogenic signaling and X-linked genetic escapees such as UTX (5, 20–22). This heightened function bolsters vaccine efficacy and improves outcomes in viral infections (e.g., HBV) and cancers (23–25). In contrast, males exhibit higher total NK cell counts—particularly the cytotoxic CD56^dim^ subset—yet often show reduced per-cell effector function, a phenomenon that may be exacerbated by aging and chronic stress (11, 26).

Recent high-dimensional studies have demonstrated that human DCs and NK cells comprise functionally distinct subsets, such as the recently defined AXL^+^DCs, CD1c^+^ DCs, and various NK differentiation states (27, 28). However, systematic studies of sex biases in subset frequencies, transcriptional signatures, and inter-subset correlations in healthy humans remain sparse, limiting mechanistic insight and translation to sex-specific immunology.

To address this, we integrated multiparameter flow cytometry, scRNA-seq, and functional assays on PBMCs from 91 healthy donors. In this study, we demonstrate that females possessed higher pDCs frequencies with enhanced ISG expression and IFN-β production, while males showed more Axl⁺ and CD1c⁺ DCs. Males exhibited higher monocyte frequencies driven by classical subsets with heightened inflammatory signatures, elevated ICAM-1, and stronger Th17-skewed CD4⁺ T cell proliferation. Conversely, female monocytes were enriched for antigen presentation, antiviral ISGs, and Th2 polarization. Among NK cells, males had more mature CD56^dim^ subsets, whereas females had more CD56^bright^ and CD56^neg^ subsets with enhanced signaling, higher activating receptor expression, and greater cytotoxic marker secretion. Collectively, by integrating these cellular and molecular layers, we provide a comprehensive framework that helps explain the biological basis for sex-biased disparities in infectious and autoimmune disease outcomes.

## Results

### The frequency of DCs, monocytes and NK cells in female and male individuals

**Figure 1:**
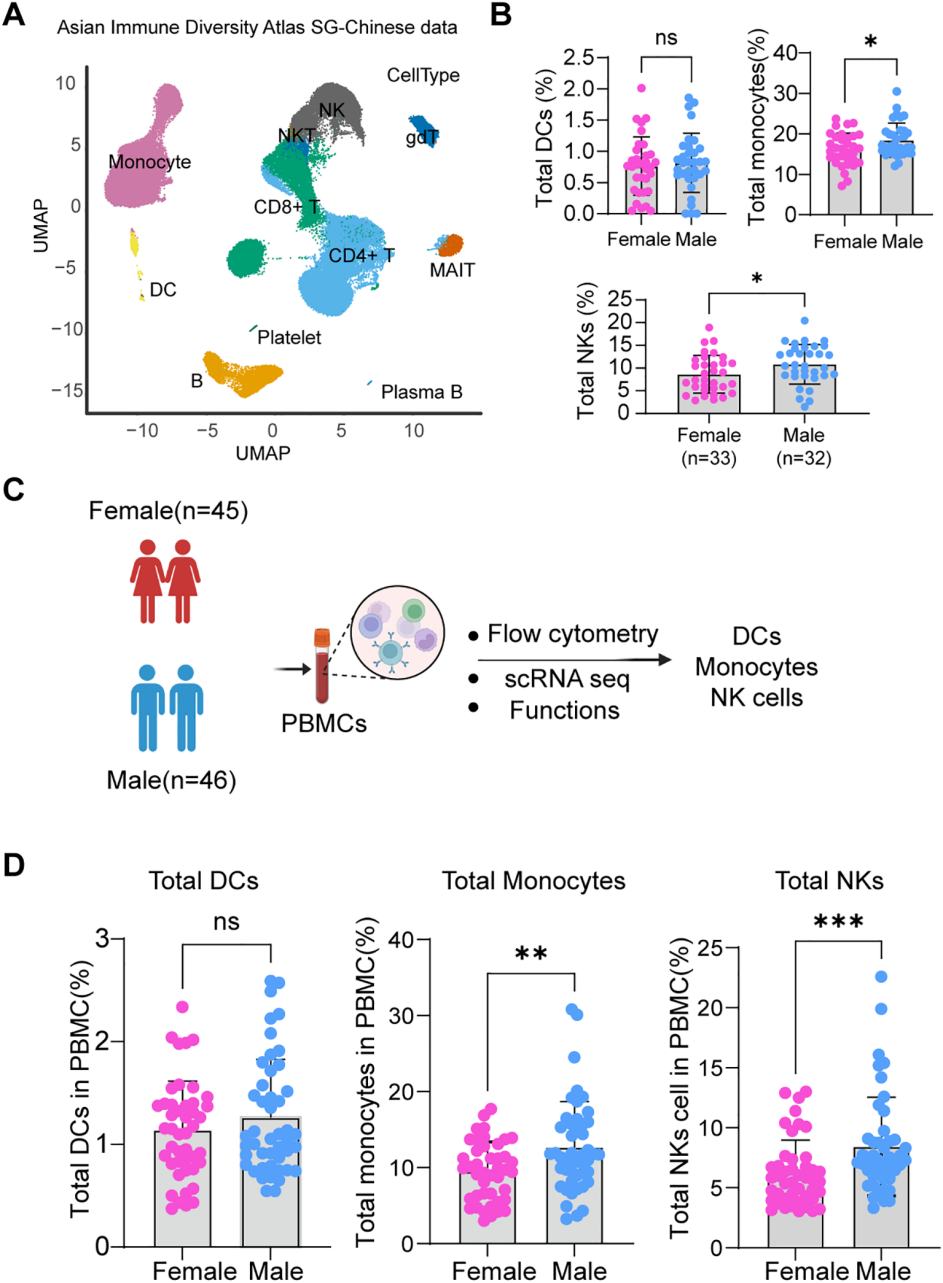
Sex-associated differences in immune cell proportions. **(A)** UMAP visualization of PBMCs from healthy donors. Eleven major immune cell types are distinguished by color: Monocytes, DCs, B cells, NK cells, Natural killer T (NKT) cells, CD4^+^ T cells, CD8^+^ T cells, Platelets, γδ T cells, Mucosal-associated invariant T (MAIT) cells, and Plasma B cells. **(B)** Comparative analysis of the relative proportions of NK cells, monocytes, and dendritic cells in PBMCs from male (n = 32) and female (n = 33) donors. **(C)** Schematic overview of the experimental design. **(D)** Comparative analysis of the relative proportions of NK cells, monocytes, and DCs in PBMCs from an independent cohort of male (n = 46) and female (n = 45) donors. Data in **(B)** and **(D)** are presented as scatter plots, with each dot representing an individual donor and data with Mean ± SD. Statistical significance was determined using the Mann-Whitney test. Significance levels are denoted as follows: ns, not significant; *p < 0.05, **p < 0.01, ***p < 0.001.

We investigated sex-associated differences in the frequency of DCs, monocytes, and NK cells using a recently published scRNA-seq dataset from the Asian Immune Diversity Atlas(AIDA) (29), focusing on Singapore Chinese individuals (**Figure 1A**; **Supplementary Table S1**). Analysis of this dataset revealed notable sex-based variation in major innate immune cell subsets. Specifically, the overall frequencies of NK cells and monocytes were significantly higher in males than in females (**Figure 1B**; **Supplementary Figures S1A–D**). To further investigate sex-based differences in innate immune composition from mainland Chinese population, we performed integrated immune profiling of peripheral blood mononuclear cells (PBMCs) from 91 healthy donors (45 females, 46 males) using multiparameter flow cytometry, transcriptomic analysis, and functional assays (**Figure 1C**; **Supplementary Table S2**). Major innate subsets—dendritic cells (DCs), monocytes, and NK cells—were quantified by flow cytometry using standardized gating strategies (**Supplementary Figure S2**). Consistent with the scRNA-seq data, we observed significantly higher frequencies of NK cells and monocytes in males. Although DC frequencies also tended to be higher in males, this difference did not reach statistical significance (**Figure 1D**). These flow cytometry-based findings were recapitulated in an independent Japanese population from AIDA study (**Supplementary Figures S3A, B**). Our findings are consistent with prior reports of sex-biased frequencies of NK cells and monocytes in peripheral blood (30–33), suggesting that sex-specific immune profiles are a fundamental biological phenomenon. However, prior studies have largely focused on bulk population-level comparisons, often overlooking subset-specific heterogeneity and recently defined cellular populations. The present study addresses this gap by providing a more granular analysis of innate immune subsets, thereby contributing to a more comprehensive understanding of sex-associated immune variation.

### Sex-specific differences in DC subset composition and gene expression

**Figure 2:**
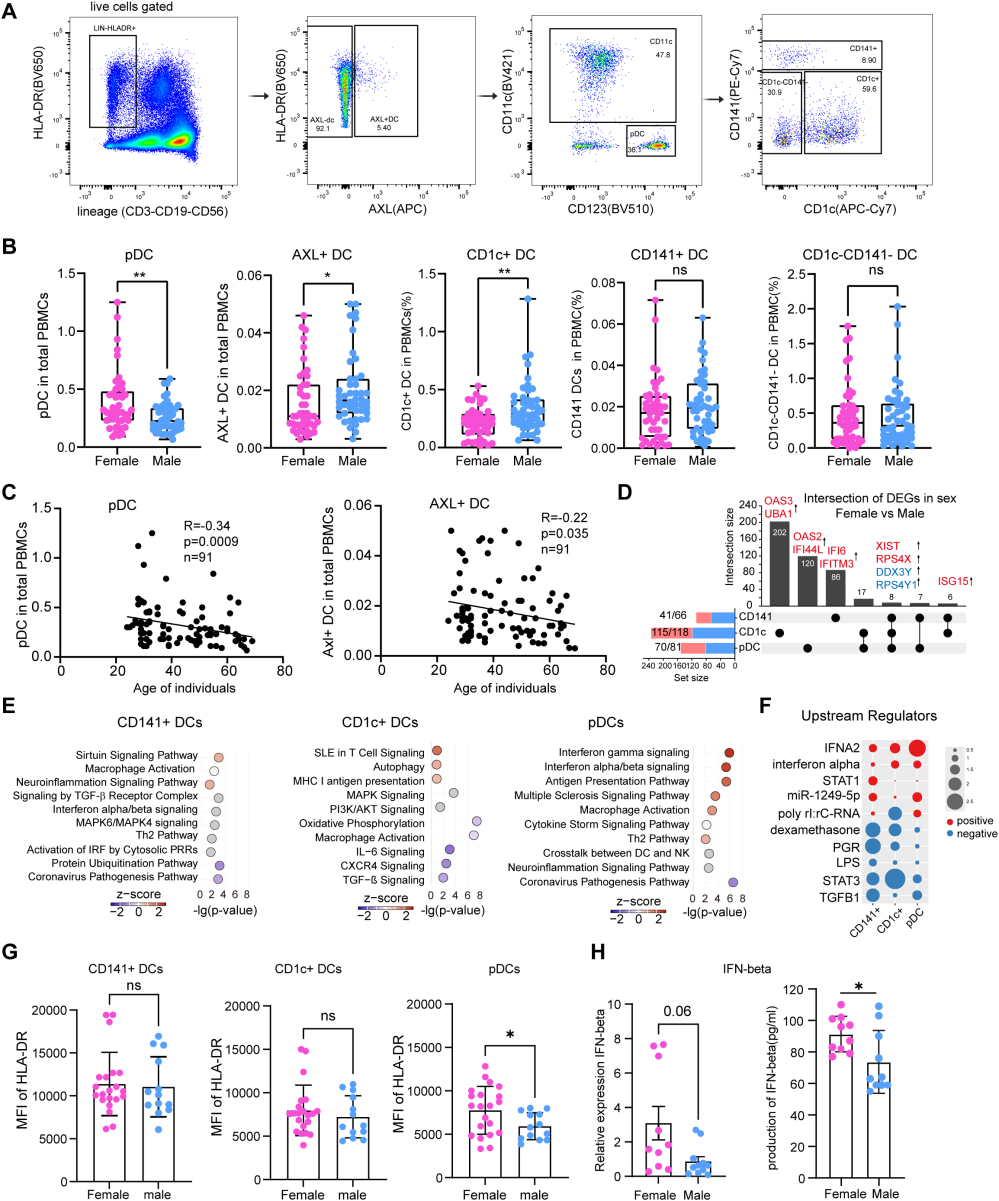
Frequency and functions comparisons of DC subsets in females and males. **(A)** Representative flow cytometry gating strategy for the identification of human DC subsets. Home-made lineage markers included CD3, CD19, CD56. AXL^+^ DC: lineage^-^HLA-DR^+^AXL^+^; pDCs: lineage^-^HLA-DR^+^AXL^-^CD11c^-^CD123^+^; CD141^+^DC: lineage^-^HLA-DR^+^AXL^-^CD123^-^CD11c^+^CD1c^-^CD141^+^; CD1c^+^DC: lineage^-^HLA-DR^+^AXL^-^CD123^-^CD11c^+^CD141^-^CD1c^+^. **(B)** Comparative analysis of the relative frequencies of DC subsets (AXL^+^DC, pDCs, CD1c^+^ DC, CD141^+^ DC, and CD1c^-^CD141^-^ DC) in PBMCs between males (n = 46) and females (n = 45). **(C)** Correlation between donor age and the frequency of pDCs (left) and AXL^+^ DCs (right). Each dot represents an individual donor. **(D)** The UpSet plot illustrating the integrated comparative analysis of differentially expressed genes (DEGs) in DC subsets between females and males. **(E)** Functional pathway enrichment analysis of DEGs in CD141^+^ DC, CD1c^+^ DC, and pDCs, as determined by Ingenuity Pathway Analysis (IPA). The color scale represents the activation z-score, with red indicating pathway activation and blue indicating inhibition. Gray indicates that an activation state could not be determined. **(F)** Predicted upstream regulators of the DEGs in each DC subset from females compared to males. The color scale represents the activation z-score, with red indicating predicted activation and blue indicating predicted inhibition of the regulator in females. **(G)** Mean Fluorescence Intensity (MFI) of HLA-DR on CD141^+^ DC, CD1c^+^ DC, and pDCs, comparing male and female donors. **(H)** Relative expression of IFN-β in R848-stimulated pDCs from females (n=10) and males (n =10), measured by qPCR, and IFN-beta secretion into the supernatant, quantified by ELISA. Data was shown as mean ± SD. Statistical significance for comparisons between two groups was determined using the Mann-Whitney test. Correlations in **(C)** were assessed by Spearman’s rank correlation. Significance levels are denoted as follows: ns,not significant; *p < 0.05, **p < 0.01.

Among the well-documented sex differences in DCs, a consistent observation is the higher frequency of pDCs in females compared to males in humans (34, 35). However, sex-associated differences across other DC subsets remain poorly characterized. In this study, we systematically analyzed five DC subsets (36, 37), including pDCs, CD1c^+^ DCs, CD141^+^ DCs, CD1c^-^CD141^-^ DCs, and Axl^+^ DCs (**Figure 2A**; **Supplementary Figure S2B**). In line with previous studies (34, 35), we observed a significantly higher frequency of pDCs in females than in males. Conversely, Axl⁺ DCs and CD1c⁺ DCs were more abundant in males, while no sex differences were detected for CD141⁺ DCs or CD1c⁻CD141⁻ DCs (**Figure 2B**). Age stratification revealed that middle-aged males had higher CD1c⁺ DC frequencies than middle-aged females, with a similar but non-significant trend in the young group (**Supplementary Figure S4A**). Conversely, pDCs frequencies were higher in young females than young males, and this difference persisted in middle age. Additionally, both pDCs and Axl⁺ DC frequencies declined with age in both sexes (**Figure 2C**).

To explore molecular correlates of these phenotypic differences, we performed transcriptomic profiling of sorted DC subsets (**Supplementary Figure S4B**). As expected, sex-chromosome genes such as XIST, RPS4X, DDX3Y and RPS4Y1 exhibited sex-biased expression patterns. Notably, the interferon-stimulated gene ISG15 was consistently upregulated across all three major DC subsets in females, suggesting enhanced basal antiviral preparedness (**Figure 2D**). Among the subsets analyzed, CD1c⁺ DCs displayed the highest number of differentially expressed genes (DEGs) between sexes (**Figure 2D**). In females, genes associated with antigen presentation pathways—including MHC class I processing and autophagy—were upregulated, whereas inflammation-related pathways such as IL-6 signaling and coronavirus pathogenesis were downregulated (**Figure 2E**; **Supplementary Tables S4**, **S5**). In pDCs, female donors showed enrichment of interferon-γ and interferon-α/β signaling pathways which consistent with pDCs gene signature from Japanese population in AIDA study (**Figure 2E**, **Supplementary Figure S3C**; **Supplementary Table S6**). Upstream regulator analysis further supported these findings, indicating enhanced activity of interferon-related regulators and reduced activity of pro-inflammatory regulators in female DCs (**Figure 2F**; **Supplementary Table S7**).

To functionally validate these transcriptional observations, we assessed pDCs activation capacity. Upon stimulation, female pDCs exhibited higher surface expression of HLA-DR and produced significantly more IFN-β than their male counterparts (**Figures 2G, H**; **Supplementary Table S8**). These results are consistent with prior studies and corroborate the enhanced type I interferon response potential in female pDCs at both transcriptional and functional levels.

### Sex-specific frequencies and transcriptional signatures of monocyte subset

**Figure 3:**
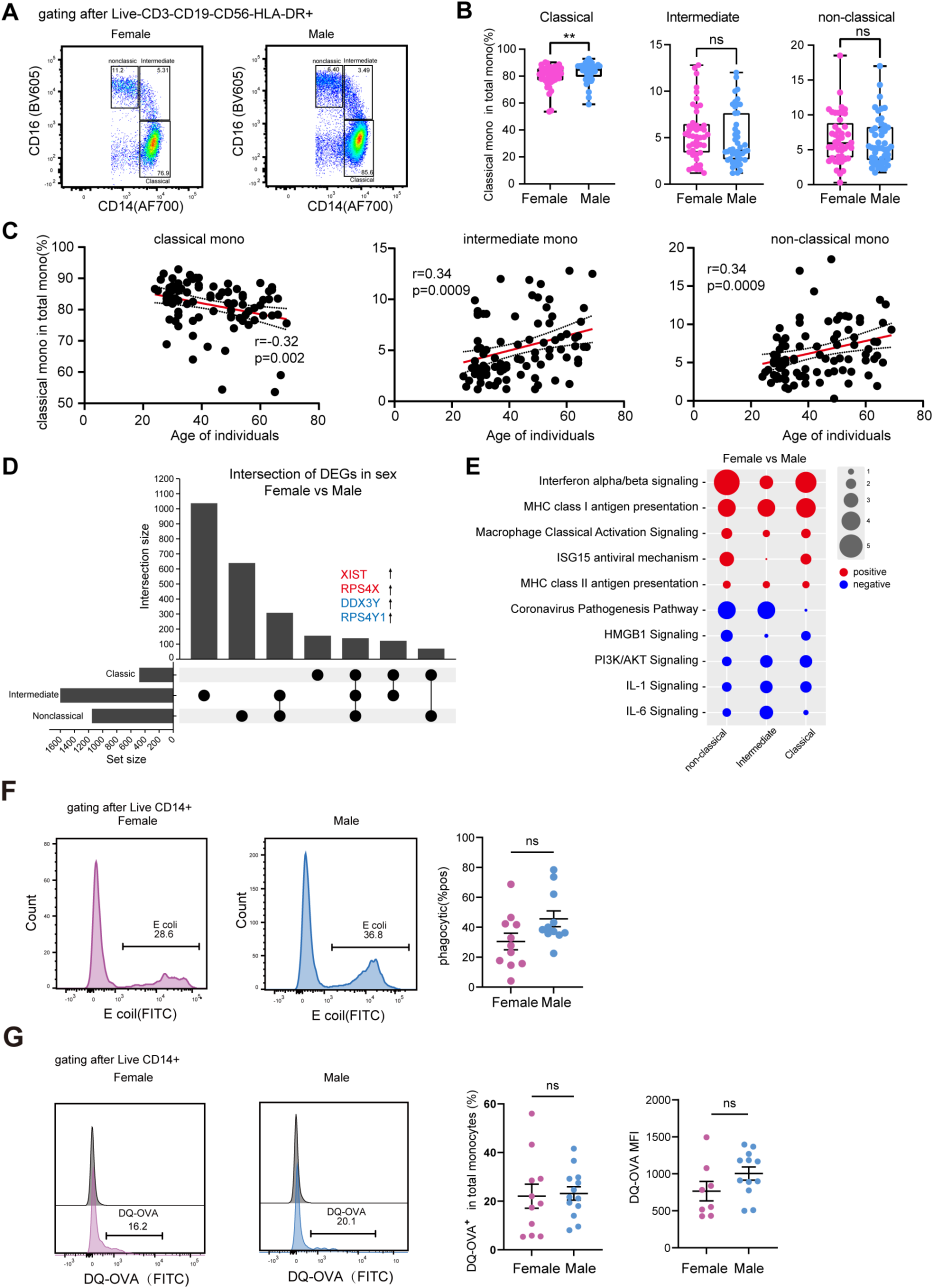
Comparative analysis of monocyte subsets in females and males. **(A)** Representative flow cytometry gating strategy for the identification of classical (CD14^++^ CD16^-^), intermediate (CD14^++^ CD16^+^), and non-classical (CD14^+^ CD16^++^) monocyte subsets. **(B)** Comparative analysis of the relative frequencies of classical, intermediate, and non-classical monocytes within PBMCs from male (n = 46) and female (n = 45) donors. Data was shown as mean ± SD. **(C)** Correlation between donor age and the frequency of classical (left), intermediate (middle), and non-classical (right) monocytes. Each dot represents an individual donor. **(D)** UpSet plot illustrating the overlap and comparison of differentially expressed genes (DEGs) identified in each monocyte subset from female versus male donors. **(E)** Functional pathway enrichment analysis of DEGs in classical, intermediate, and non-classical monocytes. The color scale represents the activation z-score, with red indicating pathway activation and blue indicating inhibition in females relative to males. The size of the circle indicates the z-score. **(F)** Phagocytic capacity of monocytes assessed using pHrodo Green E. coli BioParticles. Purified CD14⁺ monocytes from healthy female (n=11) and male (n=11) donors were incubated with pHrodo Green E. coli BioParticles for 60 min at 37 °C. pHrodo fluorescence was measured by flow cytometry to quantify phagosomal uptake. Representative histograms for female and male were shown. Quantification as percentage of pHrodo Green; each symbol represents one donor. Data was shown as mean ± SEM. **(G)** Antigen processing capacity of monocytes assessed using DQ-OVA. Purified CD14⁺ monocytes from healthy female (n=11) and male (n=11) donors were incubated with DQ-OVA (0.5µg/mL) for 3h at 37 °C. DQ-OVA fluorescence percentage of MFI were measured by flow cytometry. Data was shown as mean ± SEM. Statistical significance was determined using the Mann-Whitney test. ns, not significant; **p < 0.01.

In addition to dendritic cells, monocytes from females exhibit distinct functional and molecular profiles compared to those from males. Flow cytometry analysis revealed that classical monocytes are more frequent in males than in females, a difference that is more pronounced in younger individuals (**Figures S3A, B**; **Supplementary Figure 5A**). Of note, the frequency of classical monocytes was negatively correlated with age; in contrast, intermediate and non-classical monocytes showed the opposite trend (**Figure 3C**). Transcriptional analysis further demonstrated that female monocytes display elevated expression of genes associated with antigen presentation and interferon signaling pathways (**Figures 3D, E**, **Supplementary Figure S5B**; **Supplementary Table S9**). Specifically, female monocytes exhibited higher expression of MHC class I and II genes, proteasome-related genes, and interferon-stimulated genes (ISGs) such as IFI6, IFITM3, MX1, and BST2 across all subsets (**Supplementary Figures S5C–E**; **Supplementary Table S10**). These ISGs are known to restrict viral infections, such as HIV-1 and SARS-CoV-2 (38–40), suggesting a stronger antiviral predisposition in females. In contrast, male monocytes displayed heightened inflammatory signatures, particularly in IL-1 and IL-6 signaling pathways (**Figure 3E**). To functionally validate these transcriptional findings, we next assessed monocyte antigen uptake and processing in female and male individuals. No significant differences were observed in phagocytic function, as measured by pHrodo Green E. coli particle uptake (**Figure 3F**). Similarly, overall antigen processing—determined by the internalization of fluorescent OVA or degradation of DQ-OVA—did not differ between monocytes isolated from females and males, despite higher HLA-DR expression in female monocytes (**Figure 3G**; **Supplementary Figure S5F**). To further explore these findings, we performed pathway enrichment analysis using the Japanese dataset from the same AIDA study. The results demonstrated that monocyte subsets exhibited differential enrichment of antigen presentation pathways: classical monocytes showed marginal upregulation, whereas non-classical monocytes displayed downregulation of these pathways (**Supplementary Figure S3D**). One possible explanation for the observed discrepancy between transcriptional upregulation and functional readouts may be that post-transcriptional regulatory mechanisms modulate the efficiency of antigen presentation independently of receptor expression levels.

### Sex differences in monocyte inflammatory profile and induction of T cells

**Figure 4:**
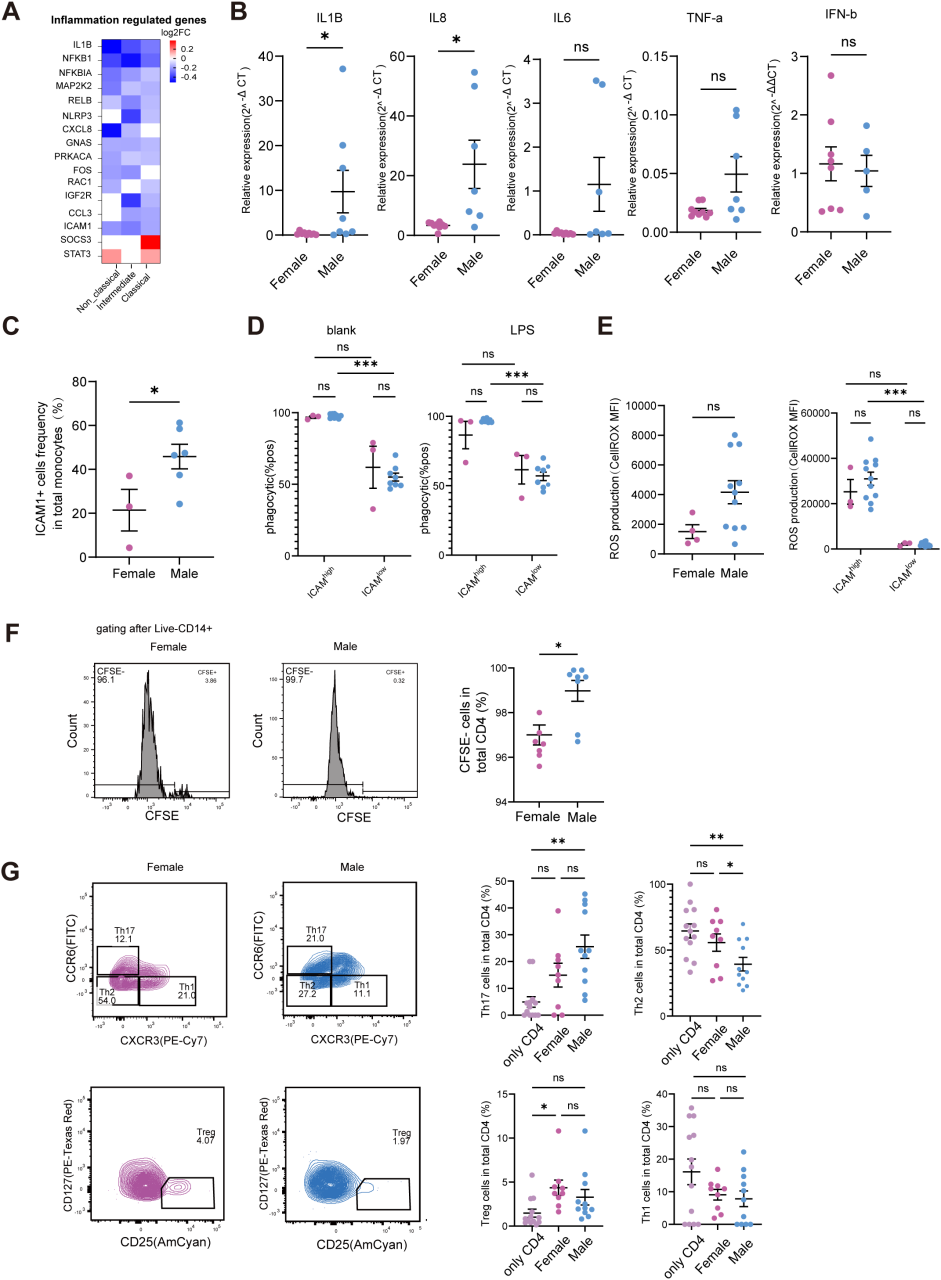
monocyte functions differ between female and male. **(A)** Fold changes of inflammation-regulated genes in females and males. Colors intensity indicate the fold changes of each gene. Red indicates higher expression in female and blue indicates low expression in female monocyte. **(B)** Expression of IL-1B, IL-8, IL-6, TNF-α and IFN-β in monocytes from male and female donors. monocytes were isolated from peripheral blood of healthy male and female donors. Gene expression was normalized to a housekeeping gene GAPDH. **(C)** Comparative analysis of ICAM-1 (CD54) expression on monocytes from male and female donors. Cell surface expression of ICAM-1 was assessed by flow cytometry. Each symbol represents an individual donor. **(D)** ICAM-1 expression was divided into high and low population, and phagocytosis was compared between female and male donors in unstimulated and LPS stimulated condition. Each symbol represents an individual donor. **(E)** Monocytes from healthy male and female donors were stimulated with LPS and assessed for ROS production by CellROX staining. Comparison of ROS levels (MFI) between male and female monocytes. Also, ICAM-1^high^ and ICAM-1^low^ subsets based on surface ICAM-1 expression. ROS production was compared between these subsets within each sex. **(F)** Mixed lymphocyte reaction (MLR) was performed by co-culturing CFSE-labeled allogeneic naïve CD4^+^ T cells with monocytes isolated from healthy female and male donors. T cell proliferation was assessed by flow cytometry based on CFSE dilution. Representative histograms show CFSE fluorescence profiles of T cells co-cultured with monocytes. **(G)** Naïve CD4^+^ T cells were co-cultured with monocytes from female or male donors for 5 days. T helper subset frequencies were determined by flow cytometry using surface markers: CCR7, CCR10, CD183, CD194, CCR6, CD25 and CD127. Data show the percentage of each subset within CD4^+^ T cells. Each symbol represents an individual donor; bars indicate mean ± SEM. Statistical significance was determined using the Mann-Whitney test. ns, not significant; *p < 0.05, **p < 0.01, ***p< 0.001.

We next assessed inflammatory responses using transcriptome analysis, which revealed that monocytes from males exhibited slightly higher expression of inflammatory genes compared to those from females (**Figure 4A**). Consistently, we found that IL-1B and IL-8 were higher expressed in monocyte from male compared with from female (**Figure 4B**). Among these, ICAM-1 (also known as CD54)—a cell surface glycoprotein critical for immune cell interactions and inflammatory responses—was significantly elevated in male monocytes at the transcriptional level (**Figure 4A**). Consistently, flow cytometry analysis confirmed that ICAM-1 protein expression was also higher in male monocytes (**Figure 4C**). Previous studies have linked higher ICAM-1 expression to enhanced functional outcomes, including bacterial phagocytosis and reactive oxygen species (ROS) production (41, 42). Consistent with this, ICAM-1^high^ monocytes displayed greater phagocytic capacity than ICAM-1^low^ counterparts. However, no significant difference in phagocytosis was observed between male and female monocytes overall (**Figure 4D**). Similarly, while ICAM-1 expression influences ROS production, we found no statistically significant difference in baseline ROS levels between female and male monocytes. Nevertheless, upon LPS stimulation, ICAM-1^high^ monocytes from both genders exhibited markedly higher ROS production compared to ICAM-1^low^ monocytes (**Figure 4E**).

To assess the functional capacity of monocytes in T cell activation, we performed a mixed lymphocyte reaction (MLR) assay using allogeneic naïve CD4^+^ T cells. Our results demonstrated that monocytes from males induced stronger CD4^+^ T cell proliferation compared to those from females (**Figure 4F**). This enhanced T cell stimulatory capacity may be attributed, at least in part, to the higher ICAM-1 expression on male monocytes, as ICAM-1 plays a critical role in stabilizing the immunological synapse during antigen presentation. Additionally, T cell polarization assays revealed distinct functional outcomes: monocytes from females promoted a higher frequency of Th2 cells, whereas monocytes from males showed a trend toward increased Th17 cell differentiation (**Figure 4G**). These sex-dependent differences in T cell polarization may have important implications for understanding the disparities in immune-mediated diseases, with females being more prone to Th2-dominated conditions such as asthma and allergies, while males exhibit higher susceptibility to certain inflammatory and autoimmune disorders associated with Th17 responses.

### Female NK cell subsets show more effector functions than male NK cells

**Figure 5:**
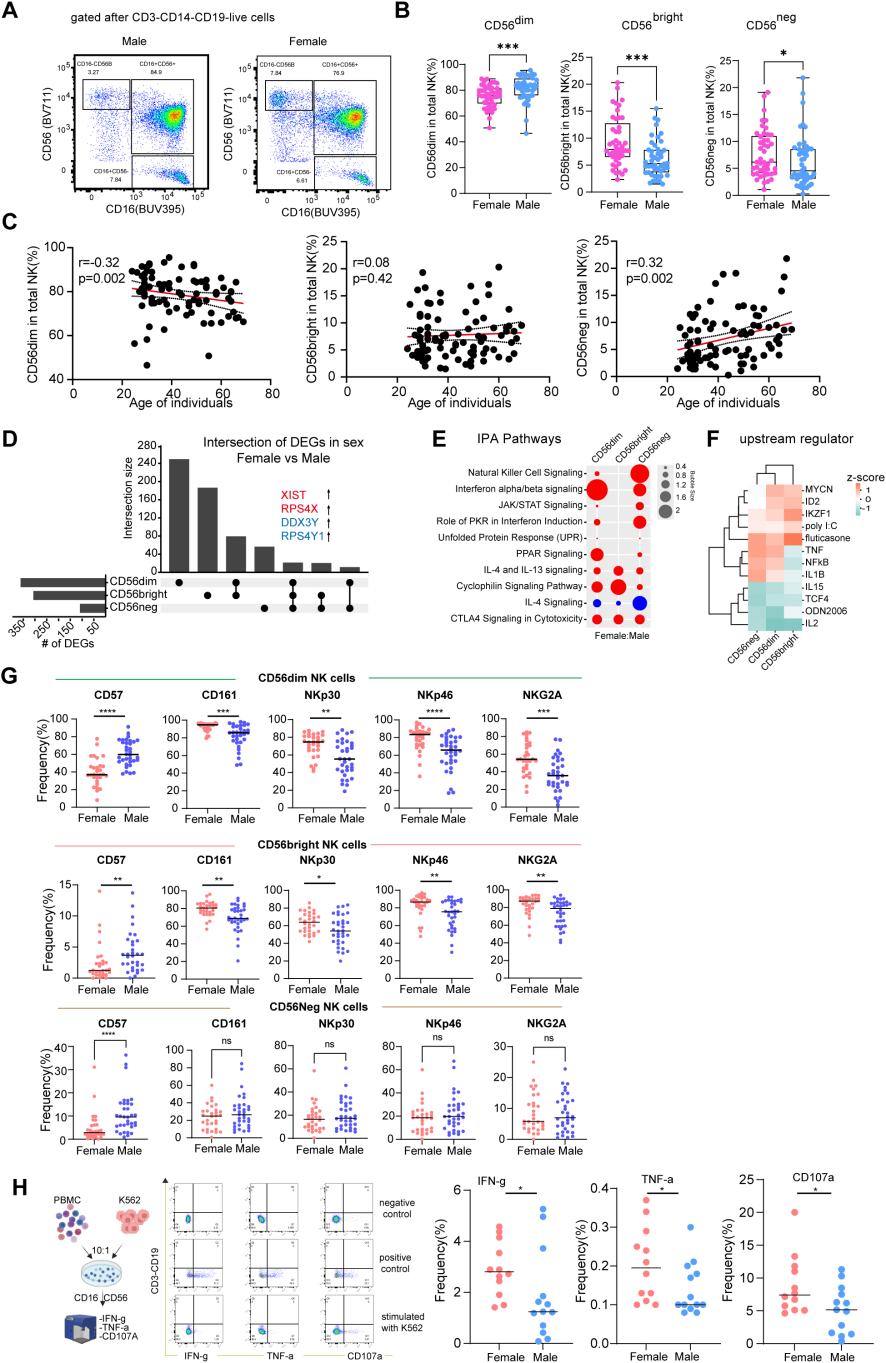
Sex-associated changes in NK cell subsets and functional profiles. **(A)** Representative flow cytometry gating strategy for the identification of CD56^dim^, CD56^bright^, and CD56^neg^ NK cell subsets within total lymphocytes. **(B)** Comparative analysis of the relative frequencies of NK subsets CD56^dim^, CD56^bright^, and CD56^neg^ in PBMCs from males (n = 46) and females (n = 45). Data was shown as mean ± SD. **(C)** correlation among age of individuals and proportions of NK cell subsets (CD56^dim^, CD56^bright^, and CD56^neg^) in PBMCs. **(D)** UpSet plot illustrating the overlap and comparison of differentially expressed genes **(DEGs)** identified in NK cell subsets from female versus male donors. **(E)** Predicted functional pathways of DEGs. Red indicates pathway activation, while blue indicates pathway deactivation. **(F)** Predicted upstream regulators of the DEGs in each NK cell subset from females compared to males. The color scale represents the activation z-score, with red indicating predicted activation and blue indicating predicted inhibition of the regulator in females. **(G)** Comparative analysis expression levels of CD57, CD161, NKp30, NKp46, and NKG2A in CD56^dim^, CD56^bright^, and CD56^neg^ NK cells from males and females. Data was shown as mean ± SD. **(H)** Functional assessment of total NK cells co-cultured with K562 target cells at a 10:1 effector-to-target (E:T) ratio. Graphs show the frequency of IFN-γ, TNF-α, and CD107a NK cells from male and female donors, measured by intracellular cytokine staining. Data was shown as median. Statistical significance for comparisons between two groups was determined using the Mann-Whitney test. Correlations were assessed by Spearman’s rank correlation. Significance levels are denoted as follows: ns, not significant; *p < 0.05, **p < 0.01, , ***p< 0.001.

We next investigated NK cell subsets based on CD56 expression levels in both females and males (**Figure 5A**, **Supplementary Figure S6A**). Our analysis revealed significant sex-based differences in NK cell subset proportions. Specifically, males exhibited a higher proportion of CD56^dim^ NK cells compared to females, while the proportion of CD56^bright^ and CD56^neg^ NK cells was significantly lower in males (**Figure 5B**). These findings are consistent with a previous study conducted in the Korean population (26). Given the known influence of age on NK cell proportions, we further demonstrated that proportion of CD56^dim^ NK cells were negatively correlated with age, while the proportion of CD56^neg^ NK cells were positively correlated with age (**Figure 5C**).

To further explore transcriptomic changes, we first identified three distinct NK cell clusters, which were annotated as CD56^dim^, CD56^bright^, and CD56^neg^ subsets (**Supplementary Figures S6B, C**). We then generated UpSet plots to visualize sex-related differentially expressed genes (DEGs) (**Figure 5D**). The number of DEGs was comparable across all three NK cell subsets in both age groups. Notably, female-specific genes such as XIST, RPS4X, and PRKX were highly expressed in females, while male-specific genes, including DDX3Y and RPS4Y1, were expressed in males (**Figure 5D**), align with recent studies (43, 44). The IPA pathway enrichment analysis revealed that NK cells from females exhibited enhanced functional activity compared to those from males. Key pathways such as natural killer cell signaling, interferon alpha/beta signaling, and were relatively more activated in females (**Figure 5E**; **Supplementary Table S11**). Furthermore, IPA predicted the activation of gene signatures associated with the upstream regulators ID2 and IKZF1, both of which are critical for NK cell development and maturation (**Supplementary Table S12**) (45). This suggests potential sex-specific differences in NK cell maturation processes. In contrast, gene signatures associated with the cytokines IL-2 and IL-15, which are essential for NK cell survival and proliferation (46), were inhibited in females (**Figure 5F**). These findings suggest that males may exhibit an enhanced proinflammatory background and heightened NK-mediated immunity compared to females, both of which increase with aging. To validate the scRNA-seq findings, we examined the protein expression of key NK cell markers, including CD161, CD57, NKp30 and NKp46, and NKG2A (**Supplementary Figure S2A**). The results demonstrated that CD57 was highly expressed in CD56^dim^ and CD56^bright^ NK cells in males, while CD161 was highly expressed in these subsets in females. No differences in these markers were observed in the CD56^neg^ population, suggesting that CD56^dim^ and CD56^bright^ NK cells in females are less mature but more differentiated (**Figure 5G**). Moreover, the activating receptors NKp30 and NKp46 were more highly expressed in females, as was the inhibitory receptor NKG2A (**Figure 5G**). Functional analysis further supported these findings, demonstrating that NK cells from females exhibited higher secretion of cytotoxic markers, including IFN-γ, TNF-α, and CD107a, compared to males (**Figure 5H**). Transcriptional factors have been shown important regulate NK cells functions, we analyzed the mRNA level of T-bet, Eomes and GATA3 transcription factors but no difference in female and male NK cell (**Supplementary Figure S6E**). Our findings reveal significant sex- and age-related differences in NK cell subsets, transcriptomic profiles, and functional activity. Females exhibit enhanced NK cell signaling and activation, whereas males show increased NK cell maturation.

### Sex differences and correlation networks in immune cell subsets

**Figure 6:**
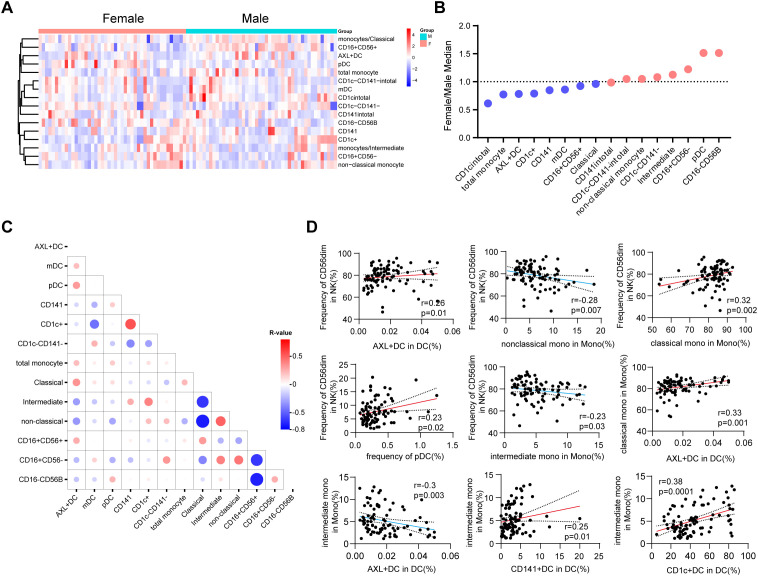
Sex differences in global immune cell composition. **(A)** Heatmap displaying all flow cytometry parameters analyzed in this dataset. The colored bar at the top of the heatmap indicates the study cohort. **(B)** Ratios of immune cell population frequencies (female/male) across the study cohort. **(C)** Correlation matrix illustrates relationships between immune cell subtypes and parameters. **(D)** correlation plot among subsets of DCs, monocytes and NK cells). Red lines indicate positive correlations, while blue lines indicate negative correlations. Spearman correlation analysis was performed.

To provide a comprehensive overview of immunological sexual dimorphism, we analyzed the frequency ratios of immune cell populations and visualized these parameters via heatmaps. Our analysis revealed distinct sex-specific signatures: CD56^bright^ NK cells and pDCs were significantly more abundant in females, whereas CD1c^+^ DCs and monocytes were more prevalent in males (**Figures 6A, B**). These findings align with previous studies in non-Chinese populations (47), suggesting that these sex-based differences in immune composition are a conserved phenomenon across diverse ethnic backgrounds. Beyond individual cell frequencies, we applied a systems immunology approach to investigate cell-cell interactions and emergent regulatory properties. Building on work by Brodin et al., who demonstrated nonrandom, reproducible correlation patterns among immune subsets (48). we examined the co-occurrence of specific cell types. We found that CD56^dim^ NK cells correlated positively with Axl^+^ DCs (r=0.26, *p* = 0.01), classical monocytes (r=0.32, *p* = 0.002), and pDCs (r=0.23, *p* = 0.02), but negatively with non-classical (r=-0.28, *p* = 0.007) and intermediate monocytes (r=-0.23, *p* = 0.03). Furthermore, classical monocytes showed a positive correlation with AXL^+^ DCs, whereas intermediate monocytes were negatively correlated with this subset (r=-0.3, *p* = 0.003). Additionally, both CD141^+^ DCs and CD1c^+^ DCs exhibited positive correlations with intermediate monocytes (**Figures 6C, D**).

## Discussion

This study provides a comprehensive analysis of the sexual dimorphism in PBMC immune composition, uncovering significant sex-based differences across several immune cell subsets, particularly in NK cells, DCs, and monocytes. Notably, data on sexual dimorphism in immune function within Asian populations, particularly the Chinese, remain limited (29, 44, 49).

We identified sex differences in DC subsets. Consistent with prior studies, our results demonstrated that the frequency and type I interferon production of pDCs were higher in females than in males, with this trend being more pronounced in younger age groups (6, 50). Estrogens enhance the expression of endosomal TLR3, TLR7, and TLR9, which are key regulators of type I interferon production. Human female pDCs, when treated with TLR7 and TLR8 agonists—but not TLR9 agonists—produced more IFNα and exhibited higher transcription of the IFN-stimulated gene IRF5 compared to pDCs from males (7, 50, 51). These findings align with recent observations from a study on individuals undergoing gender-affirming testosterone therapy, which showed that testosterone exposure attenuates pDC function (52). In contrast, males showed increased CD1c^+^ and Axl^+^ DC subsets, and transcriptome levels with enhanced antigen presentation in inflammatory contexts, though with less interferon activity. One study showed that total mDC numbers did not significantly vary with sex or age (53). our study found no difference in total mDC frequency but revealed clear sex-biased differences at the subset level, underscoring the importance of high-resolution immune profiling.

The observed increase in Axl^+^ DCs in males is particularly noteworthy, given the emerging role of this hybrid population in bridging innate and adaptive immunity. Axl^+^ DCs (also known as ASDCs), which share features of both pDCs and classical DCs, are potent drivers of T cell polarization and have been implicated in viral pathogenesis, including facilitating HIV-1 transmission and replication (27, 54). To date, no studies have specifically analyzed sex differences in Axl⁺ DC abundance, frequency, or function—likely due to their relative rarity within the total DC population, which poses significant challenges for detection and statistical power in human cohorts. One practical approach to overcome this scarcity in primary human samples is to collect larger blood volumes, such as through leukapheresis and an alternative and increasingly promising strategy involves *in vitro* generation or differentiation of Axl⁺ DCs from more abundant progenitor sources.

Monocytes also displayed marked sex differences, with males exhibiting higher classical monocyte frequencies and inflammatory gene signatures. Furthermore, the pathway analysis using IPA revealed that several Interferon-related and innate immune activation pathways were upregulated in females, including IFN signaling as the top pathway for the sex differences. These findings are consistent with previous studies showing that female monocytes are more efficient at antigen presentation and have stronger responses to viral stimuli. Of note, this appears to be conserved across a variety of species, including birds and mice (43, 55). However,

On the other hand, male monocytes displayed higher levels of inflammatory cytokines independent of chronological age, including IL-1β and IL-6, indicating a sex-dependent divergence in inflammatory responses (15, 56). Our findings are in agreement with other studies reporting a higher cytokine production response in men than in women after stimulation with gram-negative agents (17, 57). Cytokines, such as TNF-α and IL-6, production responses to sepsis are higher in men than in women, which could be one of the factors causing the higher incidence of sepsis among men (58).

A key observation in this study is the discrepancy between transcriptional profiles and functional outcomes in antigen processing and uptake. Despite elevated expression of antigen presentation-related genes in female monocytes, including higher HLA-DR surface levels, no significant sex differences were detected in phagocytic activity (assessed via pHrodo Green *E. coli* uptake) or antigen processing (measured by OVA internalization and DQ-OVA degradation). This lack of concordance suggests that post-transcriptional mechanisms—such as microRNA regulation, protein stability, or translational efficiency—may override gene expression changes to maintain functional homeostasis (59–61). Future studies could employ proteomics or single-cell functional assays to dissect these regulatory layers and determine if environmental factors, like hormonal influences (e.g., estrogen in females), modulate translation without altering phagocytosis or processing efficiency.

Regarding inflammatory profiles, the elevated ICAM-1 expression in male monocytes, validated at both transcript and protein levels, underscores a potential mechanism for sex-biased immune activation. ICAM-1 is pivotal for leukocyte adhesion and signaling, and its association with enhanced phagocytosis and ROS production in ICAM-1^high^ cells aligns with prior literature linking it to antimicrobial defense. However, the absence of overall sex differences in baseline phagocytosis or ROS, despite ICAM-1 disparities, implies compensatory mechanisms or threshold effects where ICAM-1 levels must exceed a certain point to manifest functional gains. Intriguingly, LPS stimulation unmasked heightened ROS in ICAM-1high monocytes from both sexes, suggesting that sex differences may become more apparent under inflammatory stress, such as during infection. This could explain male predisposition to hyperinflammatory states in conditions like COVID-19 or bacterial sepsis. Functionally, the superior T cell stimulatory capacity of male monocytes, as evidenced by enhanced CD4^+^ T cell proliferation in MLR assays, may stem from higher ICAM-1 facilitating stable immunological synapses. Moreover, female monocytes favoring Th2 differentiation and males trending toward Th17. These differences could be hormonally driven, with androgens promoting proinflammatory cytokines in males and estrogens enhancing anti-inflammatory or antiviral pathways in females.

Focusing on NK cells, our results uncover significant sex- and age-related disparities in subset distribution and function. Males exhibited a greater proportion of CD56^dim^ NK cells, associated with maturation and less cytotoxicity, while females showed higher CD56^bright^ NK cell activity, linked to effector functions like IFN-γ and TNF-α secretion. These findings are consistent with a previous study that showed higher CD56^dim^ NK proportions in Korean males (26, 44), and Xu et al., who reported stronger female IFN-α responses in a Chinese HBV cohort (62). Transcriptomic analysis revealed that female NK cells upregulate pathways like NK cell signaling and interferon signaling, potentially driven by X-linked genetic advantages, such as UTX escape, as reported by Cheng et al. (20). Conversely, male NK cells showed higher CD57 expression, indicating greater maturation, yet reduced activating receptor (NKp30, NKp46) expression, suggesting a trade-off between numbers and effector capacity. However, the exact mechanisms control remains unclear. Age further modulated these differences, with young females displaying more pronounced CD56^bright^ activity, a trend that diminished in middle-aged groups, aligning with a previous study on age-sex interactions in NK function (21). These results suggest females may possess a more responsive NK-mediated innate immunity, while males lean toward a mature, proinflammatory NK profile that intensifies with aging, potentially influencing infection and cancer outcomes.

Interleukin-15 (IL-15) is a pleiotropic cytokine critical for the development, homeostasis, and functional activation of NK cells. Monocytes serve as an important cellular source. Upon stimulation by pathogens or inflammatory signals, monocytes produce IL-15 both as a membrane-bound form and as a soluble cytokine, which signals through the IL-15 receptor complex (IL-15Rα/IL-2Rβ/γc) expressed on NK cells. This interaction promotes NK cell survival, proliferation, and cytotoxic activity, including enhanced production of effector molecules such as granzyme B and perforin, as well as increased interferon-gamma (IFN-γ) secretion (46, 63, 64). IL-15 also plays a key role in the generation and maintenance of memory-like NK cells, which exhibit enhanced responses upon re-stimulation (65). Conversely, deficiencies in IL-15 signaling result in profound NK cell deficiencies and impaired immune surveillance against viral infections and tumors (46, 66). Thus, monocyte-derived IL-15 represents a critical axis for innate immune activation, linking myeloid sensing of pathogens to NK cell-mediated effector functions. In the context of sex differences, emerging evidence suggests that IL-15 expression and signaling may be modulated by sex hormones, potentially contributing to the enhanced NK cell activity observed in females (67).

In addition to monocytes, NK cells, and dendritic cells (DCs), neutrophils also exhibit sexual dimorphism, with males demonstrating attenuated type I interferon signaling and a more pronounced pro-inflammatory profile (68, 69). These differences have been observed not only in PBMC samples from both humans and mice but also in recent studies of skin tissue. In the skin, females were found to harbor higher frequencies of several DC subsets—including type 1 conventional DCs (cDC1s), Langerhans cells (LCs), and CD11b^low^ type 2 conventional DCs (cDC2s)—compared to males. Notably, androgen receptor (AR) expression was not detected in skin DCs, implying that androgens may regulate these cells through indirect mechanisms. In contrast, type 2 innate lymphoid cells (ILC2s), which are abundant in the skin, were found to express higher levels of AR than other skin lymphocyte populations based on single-cell RNA sequencing data. Consistent with this, female mice possessed a greater number of skin ILC2s, which exhibited a more activated gene expression profile and produced higher cytokine levels than their male counterparts.

Previous studies illustrate the nonrandom and reproducible correlation structure among cell types (47, 48, 70). We showed that the propositions of DCs correlated with NKs. Indeed. Recent reviewers summarized that the NK cell-cDC1 axis provides novel pathways to increase immune responses (71, 72). In our study, pDCs showed a positive correlation with effector cells. pDCs are known to produce significant amounts of cytokines, especially type I interferons (73), which regulate inflammation and bridge innate and adaptive immunity. However, the mechanisms driving this correlation require further investigation.

Our work supports the concept that understanding health and disease requires the integration of the fundamental impact of sex on immune function. While our study provides a comprehensive view of sex differences in PBMCs immunity, limitations remain. First, the sample size is modest, and larger cohorts could refine statistical power, particularly for DCs where trends approached but did not reach significance. Second, while female participants were recruited during routine annual check-ups and advised to avoid visits during active menstruation, we did not collect detailed information regarding the phase of the menstrual cycle at the time of sampling. Given that hormonal fluctuations across the menstrual cycle are known to modulate immune cell function (74), this represents a potential confounding variable that we were unable to control for in our analysis. Future studies should incorporate menstrual phase tracking to more precisely characterize sex-based immune differences. Secondly, the study’s scope does not account for the HLA variability found in broader populations (75–77). Additionally, immune profile variations may differ across populations exposed to distinct environmental factors or disease burdens over time (32, 78). Another important factor not considered is the status of latent infections, such as CMV or EBV, which have been shown to have widespread effects on immune system function (53, 79, 80). We also do not further stimulate other microbial ligands such as gram-positive bacteria, fungi, or viruses to different innate cells. In conclusion, our findings highlight sex as a critical determinant of immune cell composition and function. These findings underscore the importance of considering sex as a fundamental biological variable in the design and interpretation of immunological studies, as well as in the evaluation of disease susceptibility and therapeutic outcomes.

## Materials and methods

### Study population

To avoid possible interference by other confounding factors, all participants met the strict healthy criteria. All participants were free from pregnancy, active cancer, and chronic diseases such as diabetes or hypertension. None of the donors received any vaccinations in the six months. Additionally, all study participants were local residents with no familial relationships among them. The demographic characteristics of the participants are summarized in **Supplementary Table 2**. All individuals gave written consent to donate samples for research purposes. The study was approved by the local institutional review boards and conducted in agreement with the Declaration of Helsinki.

### Sample collection, preparation, and storage

Healthy donor PBMCs were separated by Ficoll-Paque density gradient centrifugation methods (TBDscience, Tianjin, China). The viability of the PBMCs in each sample was examined by Trypan Blue staining and confirmed to be >90%. PBMCs were cryopreserved in a medium containing 10% DMSO and 90% FBS (Gibco, cat. A5256701) and stored in liquid nitrogen.

### NK, DC and monocytes immune phenotyping

The cryopreserved PBMCs sample were thawed, cell viability and number were recorded. 1x 10⁶ PBMCs were used for NK cell phenotyping. The PBMCs were first incubated with 2ul of FcR blocking reagents (Miltenyi Biotec, 130-059-901) at 4°C for 10 minutes. Subsequently, the cells were stained with a blue viability dye and antibodies against CD3, CD19, CD16, CD56, CD161, CD57, NKG2A, NKp30, and NKp46. For the DC and monocyte panel, the PBMCs were first incubated with 2ul of FcR blocking reagents (Miltenyi Biotec, 130-059-901) at 4°C for 10 minutes. Subsequently, the PBMCs were stained with a blue viability dye and antibodies against a home-made lineage cocktail (CD3, CD19, CD56) along with HLA-DR, AXL, CD123, CD11c, CD1c, CD141, CD14, CD16, and CD83 antibodies. The antibody master mix were added to each sample and incubated at 4°C for 20 minutes. Detailed antibody information is provided in **Supplementary Table S3**. The PBMCs flow samples were fixed 2% PFA (Beyotime, P0099) and analyzed by BD LSRFortessa flow cytometer in the flow core.

### NK cells intracellular cytokine staining

NK cell Intracellular Cytokine Staining (ICS) was performed with PBMCs stimulated with K562 cells. Briefly, NK cells were stimulated overnight with K562 cells with an effector-to-target ratio of 10:1, and brefeldin A (1 μg/mL; BioLegend), Monensin Solution (1 μg/mL; BioLegend), and CD107a antibody were added after 1-h culture and incubated for an additional 5 hours. PBMC Cells stimulated with PMA (2.5 μg/mL) and Ionomycin (0.5 μg/mL) served as a positive control, and R10 medium only served as a negative control. After stimulation, the cells were stained with surface antibodies against CD3, CD19, CD16, CD56, and blue viability dye at 4 °C for 20 min. Subsequently, cells were treated with a fixation and permeabilization solution according to the manufacturer’s protocol (Invitrogen). Cells were stained for 20 min at room temperature with antibodies directed to IFN-γ, CD107a, and TNF-α. Cells were then fixed by 2% PFA (Beyotime), acquired on an LSRFortessa flow cytometer (BD), and analyzed using FlowJo version v10 software (Tree Star). The proportion of cytokine-secreting cells had to be greater than 0.1% after subtraction to be considered a positive response.

### pDCs isolation and IFN-β production

The total pDCs were isolated from PBMCs using EasySep human pDCs isolation kit (Stemcell, Cat. no17977) and 900 pDCs was plated in 96U plate, cells were stimulated with 10ng/ml TLR7/8 agonist R848 compound (TLRL-R848, MCE) for 24hours. R848 (resiquimod) is a synthetic imidazoquinoline compound that functions as a Toll-like receptor 7/8 (TLR7/8) agonist. The cells were collected for qPCR and the supernatant was applied for IFN-β ELISA (ELK Bio, Wuhan).

### RNA isolation and qPCR

Total RNA was extracted using RNAprep Pure Micro Kit (DP420, Tiangen). The NanoDrop 2000 spectrophotometers were used to assess concentration and purity. Next, the isolated RNA was reverse transcribed into cDNA using HiScript III RT SuperMix for qPCR (+gDNA wiper) (R212, Vazyme). qPCR analysis was conducted using ChamQ SYBR qPCR Master Mix (Q711-02, Vazyme) in QuantStudio 7 (Applied Biosystems). The normalization of IFN-β gene expression levels was performed relative to GAPDH. The primer set was listed in **Supplementary Table S13**.

### Single-cell RNAseq analysis

Single-cell RNA sequencing data of PBMCs were obtained from Asian Immune Diversity Atlas (AIDA) (29) and the sample used in this study was listed in **Supplementary Table S1**. We utilized Seurat for initial quality control and subsequent clustering analysis. Cells were filtered based on thresholds for gene numbers (500–5000), unique molecular identifier (UMI) counts (1000–40000), and percentages of mitochondrial (<10%). After normalization and scaling of the remaining cell data, the highly variable features were identified using the “FindVariableFeatures” function. Dimensionality reduction was performed using these features, followed by cell clustering. The resulting cell clusters were visualized using the uniform manifold approximation and projection method (UMAP) and annotated by examining the expression of known marker genes. We used the “FindMarkers” function to identify genes specifically expressed in certain cell clusters or types. The difference in fold change was calculated as a ratio of expression values in females versus males and then log2 transformed. Genes with FDR of 0.1 or less were termed as differentially expressed genes. The analysis was performed using R 3.6.2.

### Pathway enrichment analysis

To determine canonical signaling pathways that contained the differentially expressed genes, we performed Canonical pathway and upstream regulator analysis using ingenuity pathway analysis (IPA; Qiagen, Redwood City, CA). Ingenuity Pathway Analysis (IPA) is a software program, which can analyze the gene expression patterns using a built-in scientific literature-based database (QIAGEN Inc.). The z-score, which is to infer the activation state of the implicated biological pathway/function, was determined by the observed gene regulation (“up” or down”) and a literature-derived direction of effect of the gene to the pathway (“activating” or “inhibiting”). Pathways with absolute |z-score| ≥ 1 and *p* < 0.05 (calculated by a right-tailed Fisher’s exact test) were considered significant.

### Monocyte isolation and LPS stimulation

Monocytes were then positively selected by CytoSinct™ CD14 Nanobeads (GenScript, cat. no L00956), as recommended by the manufacturer. Monocytes were assessed for purity by CD14-PerCP-Cy5.5 staining (BD, San Jose, CA), obtaining >95% CD14^+^ cells analyzed in a Fortessa flow cytometer (BD). For stimulation experiments, 1 × 10⁴ purified monocytes were seeded per well in 96-well flat-bottom culture plates in complete RPMI 1640 medium supplemented with 10% fetal bovine serum (FBS, Gibco), 100 U/mL penicillin, and 100 μg/mL streptomycin (Basalmedia). Cells were stimulated with 100 ng/mL lipopolysaccharide (LPS; MedChemExpress, cat. no HY-D1056) for 18 h at 37 °C. Unstimulated control cells were cultured under identical conditions without LPS. Following stimulation, cells were harvested for subsequent analyses, including quantitative real-time PCR (qPCR) and functional assays.

### RNA isolation and qPCR

Total RNA was extracted using PicoPure^®^ RNA Isolation Kit (ThermoFisher, KIT0214). The NanoDrop2000 spectrophotometers were used to assess concentration and purity. Next, the isolated RNA was reverse transcribed into cDNA using HiScript^®^ III RT SuperMix for qPCR (+gDNA wiper) (R212, Vazyme). qPCR analysis was conducted using SYBR qPCR Master Mix (AG11701, AG) in QuantStudio 7 (Applied Biosystems). Primer sequences for target genes (IL-6, IL-8, TNF-α, IL-1β) and the housekeeping gene (GADPH) were listed in **Supplementary Table S13**. All primers were synthesized by Sangon Biotech. Relative gene expression levels were calculated using the comparative Ct (2⁻ΔΔCt) method, with GAPDH serving as the endogenous reference gene. Results were expressed as fold change relative to unstimulated control samples.

### Assessment of monocytes antigen uptake and ROS production

For antigen processing, purified monocytes (1 × 10⁵ cells/well in 96-well plates) were incubated with 0.5 μg/mL of DQ-OVA (Invitrogen) for 3 h at 37 °C. Negative controls included pretreatment with 5 μg/mL of cytochalasin D (Sigma-Aldrich) for 30 min at 37 °C, followed by 3 h incubation at 4 °C. For bacteria uptake and ROS production, cells were incubated with 20 μg/mL of pHrodo Green *E. coli* BioParticles and 5uM cellROX (Thermo Fisher Scientific). In all cases, monocytes were incubated with particulate antigen for 3 h at 37 °C and cells were surface-stained with anti-ICAM-1 (CD54) Pacific Blue™ (clone HA58; Selleck Chemicals) and anti-CD14 PerCP-Cy5.5 (BD Biosciences) for 30 min at 4 °C. Flow cytometric acquisition was performed on a Fortessa cytometer (BD Biosciences), and data were analyzed using FlowJo software (version 10.8, BD Biosciences). Results were expressed as the percentage of pHrodo-positive cells and mean fluorescence intensity (MFI) of DQ-OVA or pHrodo.

### Mixed lymphocyte reaction assay and CD4 T cells polarization

For naive T cell preparation, Cryopreserved PBMCs were thawed, washed twice with PBS, labeled with CFSE (Beyotime) at 37 °C for 10 min and washed with R10 medium. Naive T cells were isolated using EasySep Human Naïve CD4^+^ T cell isolation Kit (STEMCELL Technologies). For T cell proliferation, purified monocytes were co-cultured with CFSE-labeled naive CD4⁺ T cells at a 1:5 ratio in 96-well U-bottom plates for 5 days in R10 medium supplemented with 10ng/ml recombinant human IL-2 (Novoprotein). After 5 days of co-culture, cells were harvested and stained with anti-CD3 PerCP-Cy5.5 (BD Biosciences) and anti-CD4 APC-Cy7 (BD Biosciences). For T cell polarization assay, cells were harvested and stained with the following surface antibodies at 37 °C for 20 min: BV421-CCR7 (Thermo Fisher), APC-CCR10 (BD Biosciences), PE-Cy7-CD183 (Thermo Fisher), PE-CD194 (Thermo Fisher) and FITC-CCR6 (Thermo Fisher). Subsequently, cells were stained with BV605-CD45RA (BioLegend), BV510-CD25 (BD Biosciences), and PE-Dazzle 594-CD127 (BioLegend), PerCP-Cy5.5 CD3 (BD Biosciences) and APC-Cy7 CD4 (BD Biosciences) at 4 °C for 20 min. After staining, cells were washed and analyzed by Fortessa Cytometer (BD).

### Statistical analysis

The data distribution was tested by normality testing before choosing any statistical analyses. Significance differences between the subsets were assessed using Mann-Whitney U tests and Wilcoxon rank-sum test to evaluate between-group differences. Correlations between different immune markers were determined with the Spearman test. Statistical analyses were performed with R (4.1.0) and Stata (13.0, College Station, TX), or GraphPad prism v9.

### Graphic illustration

The heatmaps, upset figures, correlation heatmaps, and bubble plots were generated using the Chiplot online tool (https://www.chiplot.online/). Bar plots and dot plots were generated by GraphPad prism v9. The flow data was analyzed by FlowJo (version v10) software (Tree Star).

## Data Availability

The original contributions presented in the study are included in the article/**Supplementary Material**, further inquiries can be directed to the corresponding author/s.
