# Supplementary figures and images for: Integrated analysis of sex differences in human dendritic cell, monocyte, and natural killer cell subsets
Source: Front Immunol. 2026 Mar 30;17:1750775. doi: 10.3389/fimmu.2026.1750775 (PMC13070752; doi:10.3389/fimmu.2026.1750775)

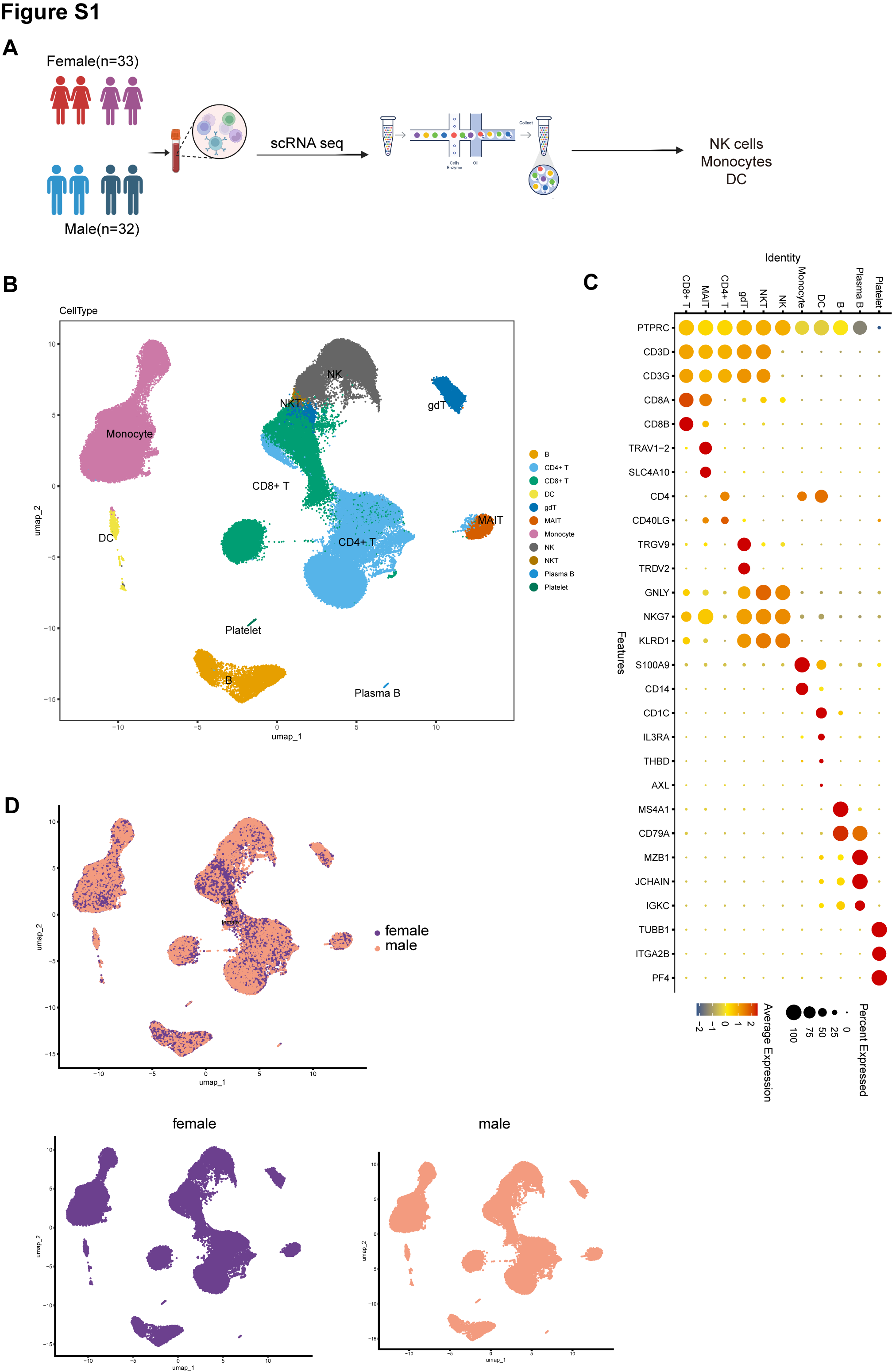

Supplement: Supplementary file 1 [file Image1.tif]

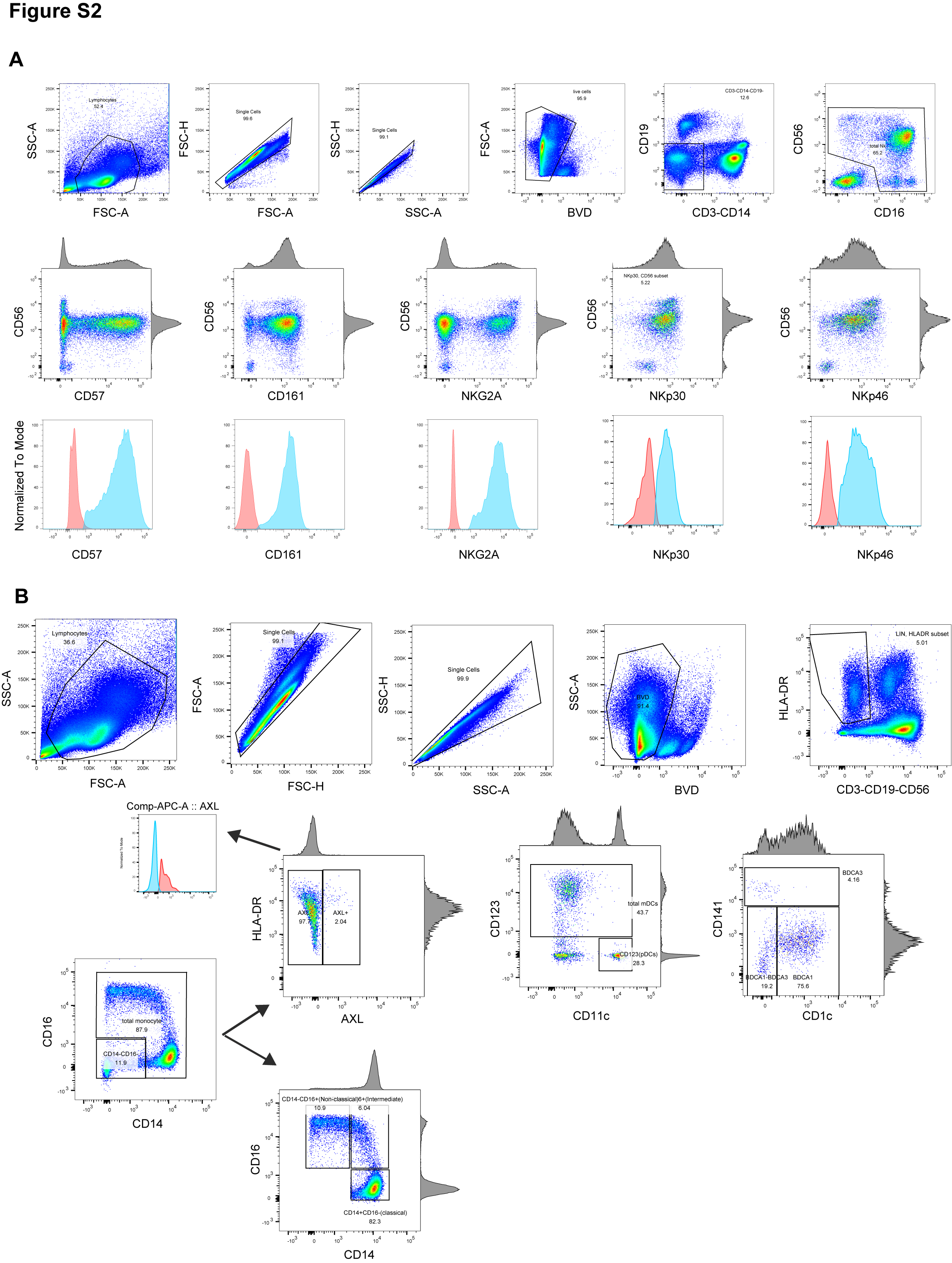

Supplement: Supplementary file 2 [file Image2.tif]

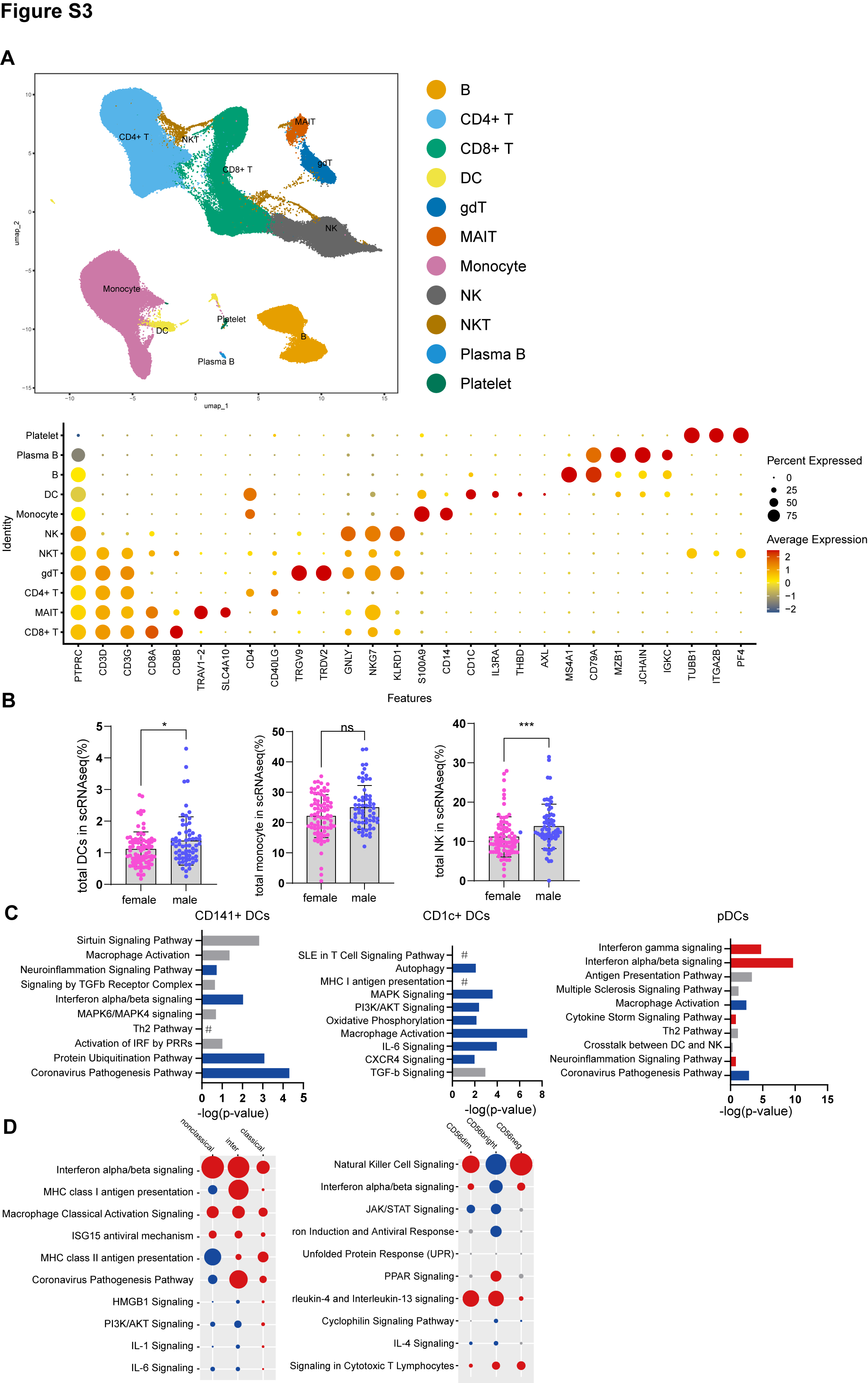

Supplement: Supplementary file 3 [file Image3.tif]

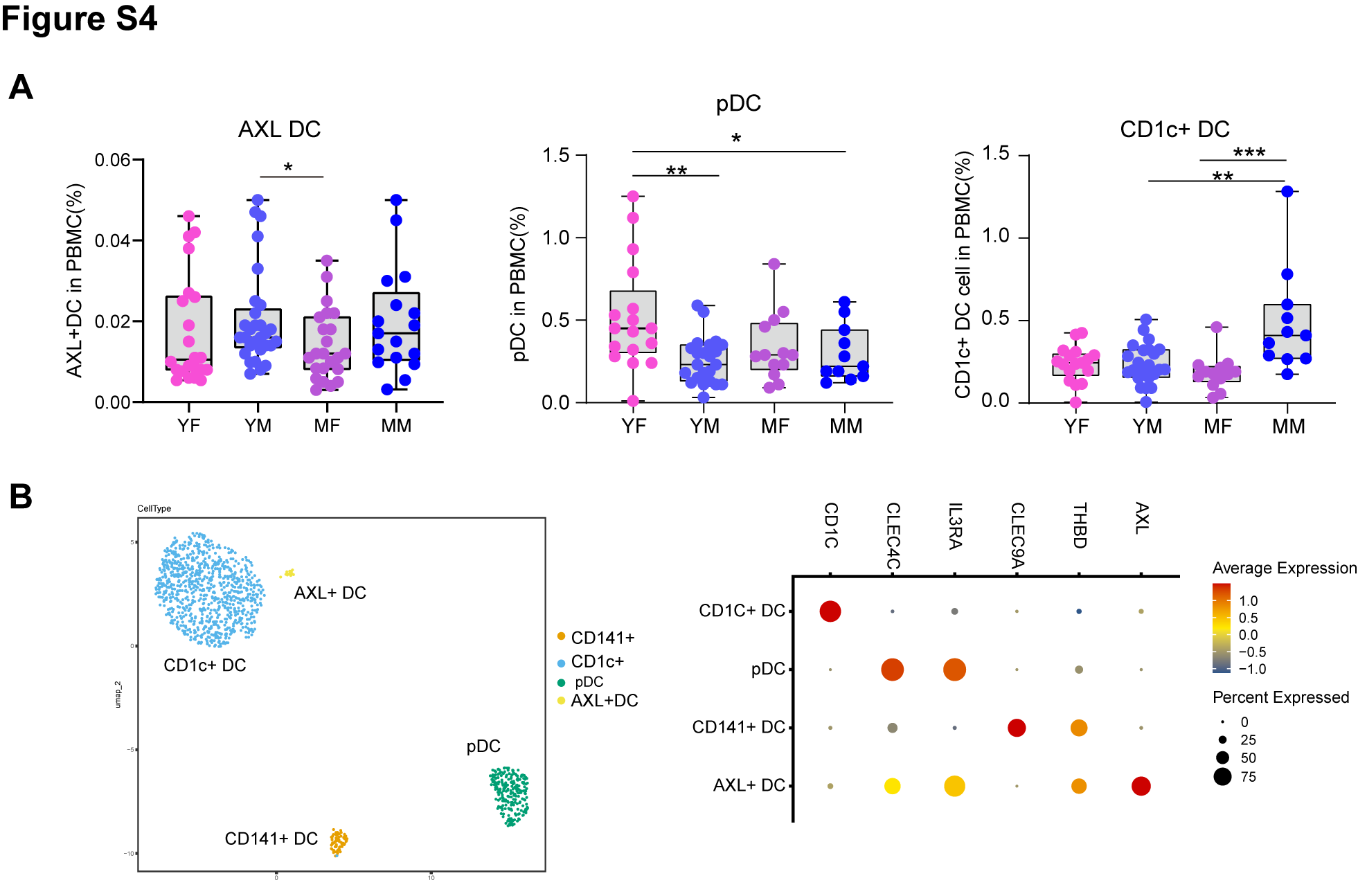

Supplement: Supplementary file 4 [file Image4.tif]

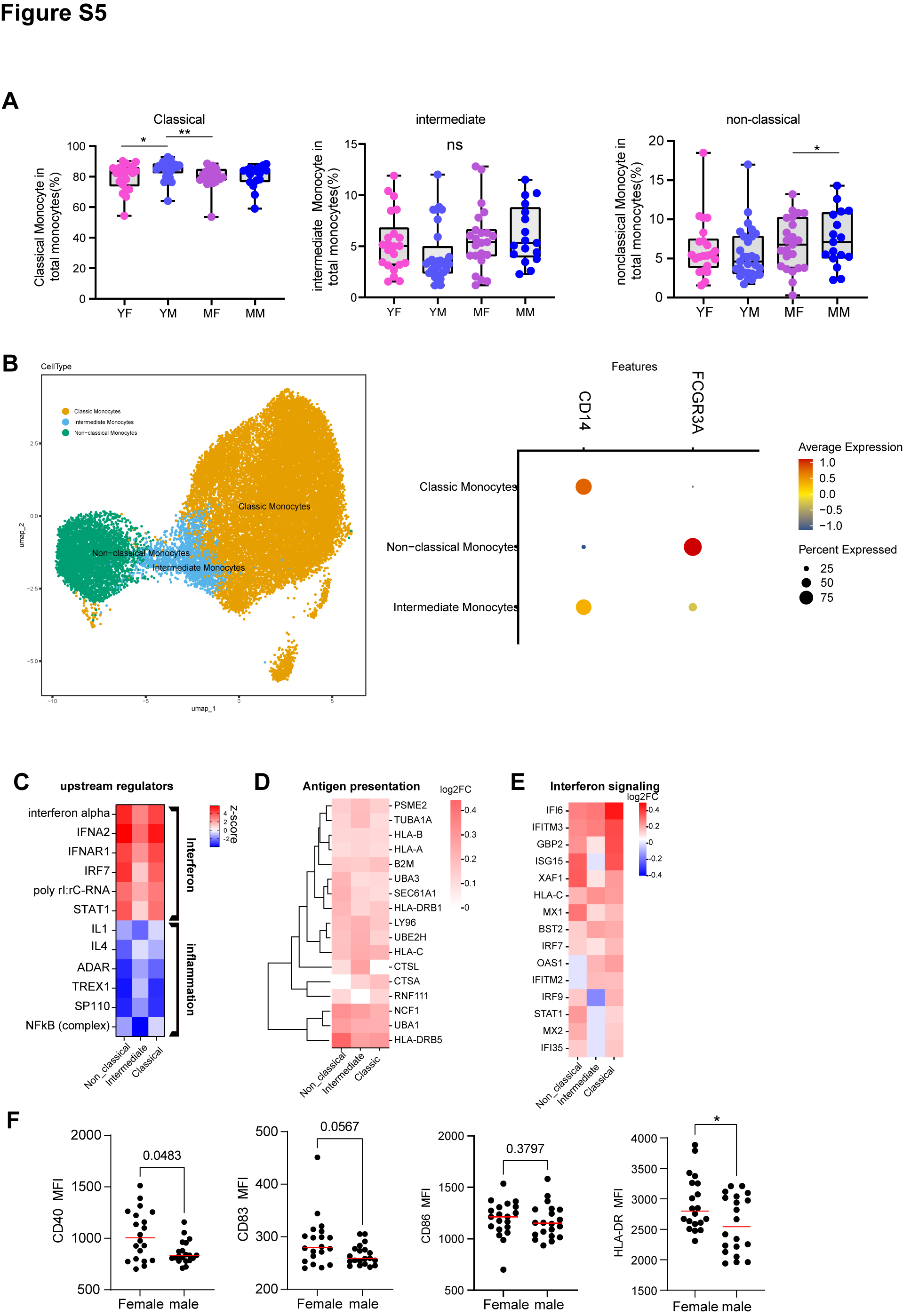

Supplement: Supplementary file 5 [file Image5.tif]

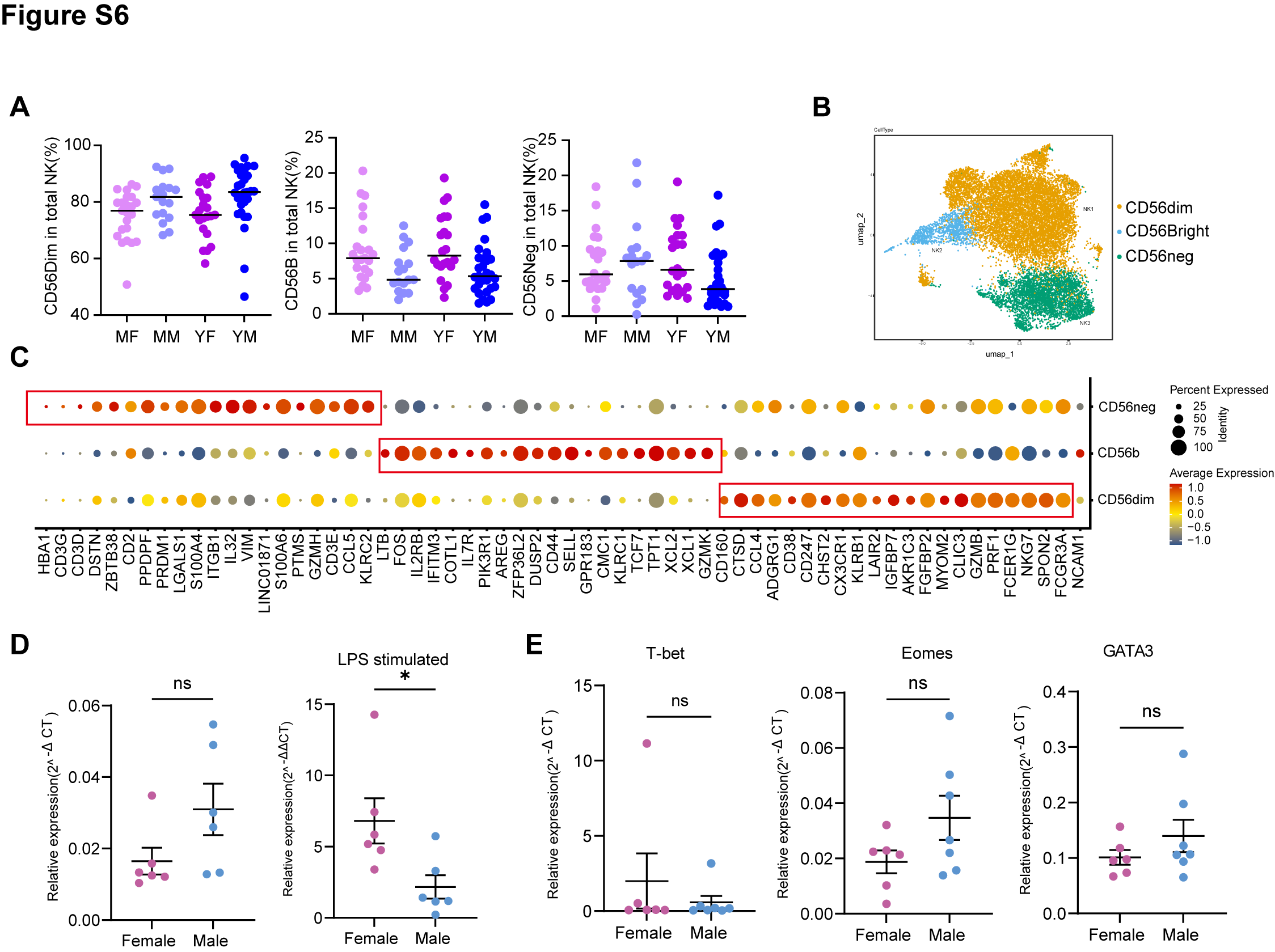

Supplement: Supplementary file 6 [file Image6.tif]
